# A Novel Simulated Annealing-Based Hyper-Heuristic Algorithm for Stochastic Parallel Disassembly Line Balancing in Smart Remanufacturing

**DOI:** 10.3390/s23031652

**Published:** 2023-02-02

**Authors:** Youxi Hu, Chao Liu, Ming Zhang, Yu Jia, Yuchun Xu

**Affiliations:** College of Engineering and Physical Sciences, Aston University, Birmingham B4 7ET, UK

**Keywords:** disassembly line balancing problem, hyper-heuristic algorithm, multi-objective optimisation, disassembly, remanufacturing

## Abstract

Remanufacturing prolongs the life cycle and increases the residual value of various end-of-life (EoL) products. As an inevitable process in remanufacturing, disassembly plays an essential role in retrieving the high-value and useable components of EoL products. To disassemble massive quantities and multi-types of EoL products, disassembly lines are introduced to improve the cost-effectiveness and efficiency of the disassembly processes. In this context, disassembly line balancing problem (DLBP) becomes a critical challenge that determines the overall performance of disassembly lines. Currently, the DLBP is mostly studied in straight disassembly lines using single-objective optimization methods, which cannot represent the actual disassembly environment. Therefore, in this paper, we extend the mathematical model of the basic DLBP to stochastic parallel complete disassembly line balancing problem (DLBP-SP). A novel simulated annealing-based hyper-heuristic algorithm (HH) is proposed for multi-objective optimization of the DLBP-SP, considering the number of workstations, working load index, and profits. The feasibility, superiority, stability, and robustness of the proposed HH algorithm are validated through computational experiments, including a set of comparison experiments and a case study of gearboxes disassembly. To the best of our knowledge, this research is the first to introduce gearboxes as a case study in DLBP which enriches the research on disassembly of industrial equipment.

## 1. Introduction

Remanufacturing is the process of rebuilding the End-of-life (EoL) products to meet the specification performance of the original manufactured products using a combination of reused, repaired and new components [1]. Remanufacturing has been widely accepted because of the enormous economic and environmental benefits as well as the great potential for sustainable development. As the inevitable process in remanufacturing, disassembly can be regarded as the reversal process of assembly, in which the EoL products are separated and retrieved for their useable and valuable sub-assemblies or components. Disassembly is traditionally carried out to retrieve valuable components using destructive or partial disassembly methods. However, in remanufacturing, the components should be disassembled non-destructively and completely in order to ensure that the remanufactured EoL products will meet the original manufacturer’s specifications [2]. Therefore, disassembly in remanufacturing requires a more precise and automated execution mode. Derivative from assembly line, disassembly line is generated as the most suitable setting for disassembly of EoL products that can manage the large-scale quantities and complex EoL products.The disassembly line balancing problem (DLBP) is assigning sequential disassembly tasks to ordered disassembly workstations to achieve better performance of some measured objectives, such as the number of stations, workload, idle time, etc. [3]. The optimisation of DLBP becomes one of the most important methods for improving the productivity and efficiency of disassembly lines by increasing efficiency and reducing cost [4].

In recent years, the volume and speed of EoL products are sharply increased because of the rapid development of technology innovation and material invention. The typical straight and single disassembly line cannot meet the requirement for dealing with massive quantities and various categories of EoL products. By considering different layout types, the overall efficiency of the disassembly line can be improved through improving the operating time of each workstation, such as two-side, U-type. The mentioned methods still have limitations that cannot deal with the multiple EoL products disassembly. Therefore, the multiple disassembly lines are proposed for dealing with multiple types of disassembly of EoL products, such as parallel disassembly lines.

Currently, the research on parallel disassembly lines is still at the initial stage. In order to improve the overall efficiency of the parallel disassembly lines, this paper has two main contributions. Firstly, a mathematical model of stochastic parallel complete disassembly line balancing (DLBP-SP) is proposed. In a remanufacturing context, remanufactured products should have the same performance specifications as the original products [5]. Therefore, the EoL products have to be completely disassembled for the subsequent remanufacturing processes. Due to the uncertain conditions of the EoL products, the disassembly time is described as stochastic numbers in this model. The optimisation objectives of the parallel complete disassembly line include the number of workstations, working load index and profits. The conflict between these optimisation objectives adds to the computational complexity of the mathematical model. Secondly, a novel simulated annealing-based hyper-heuristic algorithm (HH) is proposed to solve the multi-objective optimisation of the DLBP-SP. Partially mapped crossover and single-point insertion mutation operations are designed to satisfy precedence constraints. The search and solving space is expanded and enhanced by applying the algorithm. The effectiveness and superiority of the algorithm are demonstrated through comparison experiments. The comparison experiment is carried out based on the collected open-source dataset and proposed optimisation algorithms for validating the superiority of our proposed algorithm. In addition, the case study is implemented as a practical application in industrial disassembly. Through the case study, the stability and robustness of the proposed algorithm can also be verified. To our knowledge, this research is the first attempt to introduce the gearbox as a case study in DLBP.

The rest of this paper is organised as follows: Section 2 reviews literature from the layout type of disassembly line, optimisation algorithms for DLBP and researched disassembly products. Section 3 introduces the problem description, assumptions, notations, and mathematical model of DLBP-SP alongside an illustrated example. Section 4 introduces the novel simulated annealing-based hyper-heuristic algorithm, including explanations of the framework and detailed processes. Section 5 carries out the comparison experiment and the case study. The efficiency and performance of the proposed algorithm are analyzed and validated through the results. Section 6 gives concluding remarks and recommendations for future work.

## 2. Literature Review

The literature review covers three aspects, including layout types for disassembly lines, optimization algorithms, and the studied disassembly products of DLBP. These three aspects are the foundation for the research backgrounds, methods and objects of DLBP. The research gaps and challenges of DLBP are discussed and summarized.

### 2.1. Layout Type of Disassembly Line

**Figure 1:**
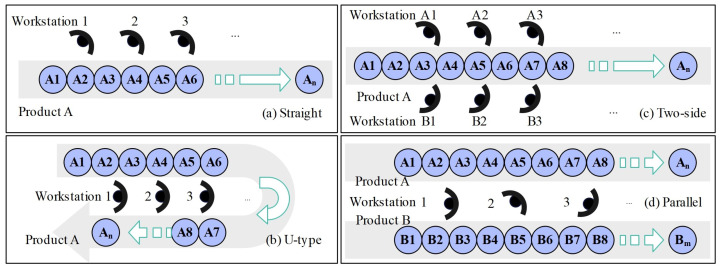
Layout type of disassembly lines.

The layout type of the disassembly line is decided at the design stage for determining the function and capability of the disassembly line, in which the number of workstations and cycle time are two key factors that affect the overall efficiency of the disassembly line. Workstations refer to any point on the disassembly line where operators execute a disassembly task on EOL products. Cycle time is the time it takes to complete each workstation task, which includes working time and idle time. The overall efficiency of the disassembly line can be improved by minimising the idle time of each workstation. Different disassembly line layouts represent different implementation modalities of EoL products disassembled by workstations. According to the literature review, there are four main layout types of disassembly lines, including straight, U-type, two-sided, and parallel, as shown in Figure 1.

The straight type is the most commonly used layout type for disassembly lines. The workstations are sequentially organized in a line array, as shown in Figure 1 [6]. The structure of the straight disassembly line is simple, making it easy to construct the mathematical model for DLBP. On this basis, several studies have incorporated different scenarios for further research, such as partial disassembly [7], and automatic robotic disassembly [8]. However, the straight disassembly system has a relatively low dynamic range and is only suitable for processing a single type of EoL product. The U-type disassembly line was first proposed by Agrawal and Tiwari [9]. Compared to the straight type, the U-type has the advantages of relatively high operation flexibility, high efficiency and short setup times [10]. The two-sided disassembly line was introduced by Wang et al. [11] and Kucukkoc [12], which is designed specifically for processing the disassembly of large-sized equipment. Both U-type and two-sided layouts cannot be used for disassembling multi-type EoL products [13]. Therefore, the parallel disassembly line was first proposed by Aydemir-Karadag and Turkbey [14] for dealing with the disassembly process of multi-type EoL products simultaneously. Wang et al. proposed the genetic simulated annealing algorithms for solving parallel DLBP under uncertainty [15]. The parallel disassembly line realizes high flexibility and can also disassemble multiple types of EoL products. With the increasing quantity and variety of EoL products, the parallel disassembly line is more appropriate and beneficial for practical application in real-world scenarios.

### 2.2. Optimization Algorithms for DLBP

The optimisation of DLBP is a typical non-deterministic polynomial (NP) complete linear programming problem [16], which cannot determine the optimal solution. According to the characteristics of DLBP, there are three main types of optimisation methods in DLBP: exact methods, heuristic algorithms and meta-heuristic algorithms. At the initial stage, the exact methods are considered and applied in DLBP. Altekin et al. [17] proposed the linear programming methods and developed the mixed integer programming formulation for solving the profit-oriented partial DLBP [18]. Igarashi et al. [19] proposed integer programming to design the disassembly system and achieve the multi-objective optimisation goals in a closed-loop supply chain. Özceylan and Paksoy [20] developed a nonlinear programming model for assigning disassembly tasks to optimize the reverse supply chain. With the development of global and intelligent optimisation algorithms, an increasing number of heuristic and meta-heuristic algorithms are introduced and applied in DLBP. The heuristic algorithms are generated and developed by imitating natural behaviours, including greedy algorithm, hill climbing algorithm, simulated annealing algorithm, ant colony algorithm, etc. McGovern and Gupta developed the mixed hill-climbing [21] and greedy algorithm [22] to generate the disassembly sequence and solve the DLBP. Kalayci and Gupta published a series of research for solving sequence-dependent DLBP, which took simulated annealing (SA) algorithm [23], and ant colony optimisation (ACO) algorithm [24]. All the heuristic algorithms are formulated and programmed with regulations for solving specific optimisation problems. Nowadays, meta-heuristic algorithms are becoming the most popular optimisation algorithm in DLBP. The meta-heuristic algorithm is derived from the heuristic algorithm, which combines the stochastic process and the local search algorithm. There are several heuristic algorithms that are also implemented to optimize the DLBP, such as the hybrid genetic algorithm (GA) [25], particle swarm optimisation (PSO) algorithm [26], artificial bee colony algorithm [27] and tabu algorithm [28] for solving the optimisation process of DLBP. Zhang et al. [29] proposed the artificial fish swarm algorithm for solving multi-objective DLBP under uncertain disassembly time. Zhu et al. [30] constructed the firefly algorithm for solving the discrete DLBP and taking hazardous disassembly operations into account.

In summary, the exact method may obtain the optimal solution, but it has limitations and is not suitable for solving the large-scale and multi-objective optimisation of DLBP. The exact method consumes high computing resources and time dealing with large-scale optimisation of DLBP [31]. According to the characteristic of heuristic algorithms, the proposed heuristic algorithms cannot obtain the optimal solution of NP problems and easily obtain the local optimal solutions [32]. The heuristic algorithms are not suitable for solving high-complexity DLBP. Meta-heuristic algorithms are able to provide more optimal solutions with limited resources, and they are suitable for dealing with large-scale and multidimensional optimization problems. However, the model and computational complexity of the meta-heuristic algorithm are higher than exact methods and heuristic algorithms.

### 2.3. The Categories of EoL Product in DLBP

Traditionally, research on DLBP is focused on constructing a mathematical model and proposing an optimization algorithm. Most case studies are implemented based on benchmark test datasets without considering actual EoL products [33]. The benchmark test datasets are commonly generated from software modelling, mainly applied for verifying and validating the performance of the proposed algorithms.

In order to promote and enrich the application of automation disassembly in the real-world, the disassembly of actual EoL products is gradually introduced in DLBP. The majority of actual EoL products are focused on waste electric and electronic equipment (WEEE), such as personal computers (PC) [34], mobile phones [35], laptops [36], etc. These WEEE products are suitable for conducting experiments due to their variety and simple physical structure. However, the resource recycling and economic benefits from the disassembly process of electronic products are limited [37]. Industrial equipment disassembly has more significant social benefits as a result of its large scale and high added value. However, only a few studies consider industrial equipment as a case study, including hammer drills [38], corn harvester cutting tables [39] and automobile engines [40].

### 2.4. Research Gaps and Challenges

In summary, there are three major research gaps and challenges according to the literature review:

1. The majority of disassembly line layout types are straight with a determined environment [41], which cannot fully model the actual disassembly scenario. Straight disassembly lines are incapable of disassembling multi-type EoL products simultaneously [42]. The mathematical model of the predetermined scenario cannot reflect the actual characteristics of both disassembly lines and EoL products.
2. The increasing complexity of the mathematical model and the uncertain conditions of DLBP limit the performance of existing optimisation algorithms. The single-objective optimisation of DLBP is linear. However, the multi-objective optimisation of DLBP-SP becomes a nonlinear and NP problem with higher computational complexity than DLBP. With the development of artificial intelligence methods, novel optimisation algorithms need to be proposed to deal with multi-objective optimization with uncertain conditions and obtain better optimisation performance.
3. The condition of EoL products is uncertain, and the disassembly sequence is also divergent. These uncertain characteristics of EoL products will lead to uncertain disassembly process sequence and time of EoL products. Most EoL products in DLBP are based on benchmark test datasets or WEEE equipment. The number of disassembly tasks of these EoL products is relatively small. The precedence constraints are relatively simple as well. Industrial equipment is another category that has great potential value for remanufacturing [37].

In accordance with the three aspects listed above, this research explores a more realistic and complex scenario of parallel assembly lines and proposes a novel optimisation model to solve multi-objective optimisation of the DLBP-SP. Furthermore, this research also introduces a new type of industrial equipment (gearbox) as a case study to enrich the disassembly research of industrial equipment.

## 3. Stochastic Parallel Disassembly Line Balancing Problem

### 3.1. Problem Description

**Figure 2:**
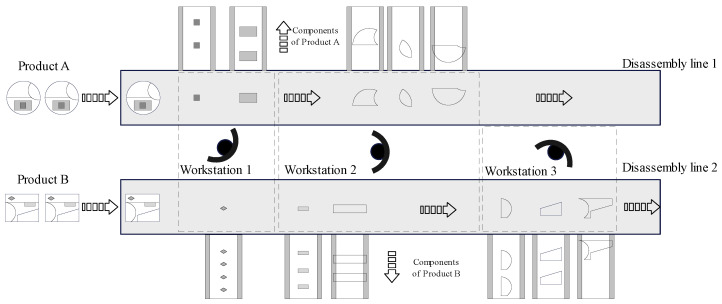
Parallel disassembly line.

The typical layout scheme of a parallel disassembly line is shown in Figure 2. There are two adjacent parallel distributed disassembly lines 1 and 2, which can simultaneously disassemble EoL products A and B, respectively. Products A and B are assumed to be layered industrial assemblies. Product A has five components and product B has six components. The darker components have greater priority to be disassembled. Furthermore, the disassembled components are delivered separately for the following remanufacturing processes. The disassembly sequence is one possible and feasible successive order for carrying out the disassembly tasks, which complies with the disassembly precedence constraints because of the restriction of the physical structure of products. Complete disassembly is the process whereby the EoL product is separated into all its components [43]. In Figure 2, both EoL products A and B are completely disassembled through workstations on disassembly lines 1 and 2, respectively.

As for the workstations, workstations 1, 2 and 3 are sequentially allocated between the parallel disassembly lines. All workstations should be capable of processing the disassembly task on one or both lines. The first two disassembly tasks of product A and the first disassembly task of product B are assigned to Workstation 1. The following three disassembly tasks of product A and the following two disassembly tasks of product B are assigned to Workstation 2. Both workstations 1 and 2 are processing the disassembly tasks on both disassembly lines. Workstation 3 is assigned the last three disassembly tasks of product B, which only works on disassembly line 2. The disassembly tasks of different EoL products are allocated to different workstations through the optimisation algorithm.

Multi-type products can be simultaneously disassembled on a parallel disassembly line. Cycle times for each disassembly line can be designed differently to improve efficiency and flexibility. Additionally, the idle time of workstations in a cycle can be reduced in order to improve their efficiency.

### 3.2. Notations and Assumptions of DLBP-SP

In this paper, we focus on the optimisation of the complete disassembly process of two different EoL products on parallel disassembly lines. In order to propose a more practical mathematical model of DLBP-SP, several related basic notations are proposed. The definition and description of notations are represented in Table 1.

Based on the basic notations, the following assumptions are pre-defined for reasoning the mathematical model of DLBP-SP.

1. Two disassembly lines are designed to be adjacent and parallel, and the workstations are located sequentially between them.
2. The cycle time of each disassembly line is pre-defined and can be different.
3. Workstations are operated by skilled workers who can work on single or both parallel disassembly lines and spend no travel time.
4. The workstations can only be allocated and process a single disassembly task at a time.
5. The precedence constraints and mean disassembly time of each disassembly task are known. Moreover, the precedence constraints of disassembly tasks should be satisfied during the disassembly process.
6. The EoL products are completely disassembled into their simplest single components. The revenue from each disassembled component is known.
7. Each disassembly task’s actual process time is stochastic, following the standard normal distribution.
8. The sum of the actual process time of assigned disassembly tasks to a workstation should not exceed the cycle time. If exceeded, the number of workstations should be added for taking the remaining disassembly tasks into new cycle time.
9. Materials and instruments are sufficient and infinite.

### 3.3. Mathematical Model of DLBP-SP

Based on the proposed notations and assumptions, the parallel disassembly line balancing with stochastic process time (DLBP-SP) is derived from the definition of the common cycle time of parallel disassembly lines, the multi-optimisation goals and the lower bound of DLBP-SP. An explanatory example is carried out to illustrate the proposed mathematical model of DLBP-SP.

#### 3.3.1. Cycle Time of Parallel Disassembly Lines

Cycle time is defined as the total elapsed time from the process beginning to the stop end of a workstation, which is pre-defined on the disassembly line [44]. According to the characteristics of parallel disassembly lines, each disassembly line ($CT_{m}$) can have the same or different cycle times. In order to manage and improve the overall performance of parallel disassembly lines, the common cycle time (CT) is adopted. Referring to Özcan [45], the modified least common multiple (LCM) method is applied in this paper. The steps of LCM method for DLBP-SP are shown below:

Step 1: Determine the LCM as the common cycle time (CT) of two different disassembly lines:

(1) $$CT=[CT_{1},CT_{2}],m=2;$$

Step 2: Calculate the coefficient values $\varepsilon_{m}$ by dividing each cycle time of the disassembly line ($CT_{m}$) by the common cycle time (CT):

(2) $$\varepsilon_{m}=CT/CT_{m};$$

Step 3: Modifying the stochastic process time of each disassembly line (${\tilde{t}}_{im}$) into parallel disassembly lines based on the coefficient values $\varepsilon_{m}$:

(3) $${\tilde{t}}_{im}=N\left(\mu_{im},\sigma_{im}\right)$$

(4) $${\tilde{t}}_{im}^{\prime }=\varepsilon_{m}\cdot {\tilde{t}}_{im}\Rightarrow {\tilde{t}}_{im}^{\prime }=N\left(\varepsilon_{m}\cdot \mu_{im},\varepsilon_{m}^{2}\cdot \sigma_{im}^{2}\right)\Rightarrow {\tilde{t}}_{im}=N\left(\mu_{im}^{\prime },\sigma_{im}^{2\prime }\right).$$

The calculated common cycle time (CT) and updated stochastic process time (${\tilde{t}}_{im}^{\prime }$) are considered in DLBP-SP. The process will be further illustrated in explanatory example.

#### 3.3.2. Multi-Objective Optimisation of DLBP-SP

In general, disassembly line balancing involves arranging the disassembly task sequence to increase overall performance. Evaluation indices can include productivity, efficiency, profit, etc. In order to validate and evaluate the optimisation performance, there are three optimisation objectives of DLBP-SP considered in this paper, including the number of workstations (*K*), workload smoothness index (*I*) and profit (*P*). The formulations for representing the multi-objective optimisation of DLBP-SP are represented as follows:

(5) $$f_{1}=\min\left(K\right)=\sum_{k=1}^{K}\max_{i=1}^{I}\left(\sum_{m=1}^{M}\sum_{j=1}^{J}x_{ijm}y_{ijmk}\right)$$

(6) $$f_{2}=\min\left(I\right)=\sqrt{\sum_{k=1}^{K}{\left(CT-T_{k}\right)}^{2}}$$

(7) $$f_{3}=\max\left(P\right)=\sum_{m=1}^{M}\sum_{i=1}^{I}r_{i}x_{ijm}-C_{s}\sum_{j=1}^{J}S_{k}-C_{p}\sum_{j=1}^{J}P_{k}-\left(CT\cdot C_{w}\right)\sum_{k=1}^{K}Z_{k}$$

(8) $$F=\min\left[f_{1},f_{2},-f_{3}\right]$$

Equation (5) represents the minimum number of workstations. Equation (6) represents the minimum smoothness index of workload. Equation (7) represents the maximum profit through the complete disassembly processes of different EoL products. Equation (8) is the multi-optimisation goal of the DLBP-SP, which achieves the minimum number of workstations, minimum working load, and maximum profit as the optimal solution. The optimisation process for multi-objectives may have mutual constraints and conflicts among objectives. Improvement of one objective’s performance will always undermine another objective’s performance. It is impossible to have an optimal solution that achieves the best performance for all objectives. In order to evaluate the optimisation results with a view to achieving the optimal performance of multi-objectives, a Pareto optimal solution (also known as a non-dominated solution) is adopted [46]. The set of Pareto optimal solutions is considered as the optimal solutions (the boundary), which none of the objectives can be improved through reducing other objectives. The above multi-objective optimisation equations must satisfy the constraint equations as follows:

(9) $$\sum_{k=1}^{K}y_{ijmk}\leq 1,\forall i\in I,j\in J$$

(10) $$\sum_{m=1}^{M}\sum_{i=1}^{I_{m}}x_{ijm}y_{ijmk}\geq 1,\forall k=1,2,\ldots ,K$$

(11) $$y_{ijmk}\leq \sum_{o=1}^{k}y_{ijmo},\forall i\in I,k\in K,o\in PAND\left(i\right)$$

(12) $$y_{ijmk}\leq \sum_{o=1}^{k}\sum_{o\in OR(i)}y_{ijmo},\forall i\in I,k\in K,o\in OR\left(i\right)$$

(13) $$\sum_{m=1}^{M}\sum_{k=1}^{K}\sum_{i=1}^{I}T_{k}x_{im}\leq CT,\forall i\in I,j\in J,k\in K$$

(14) $$x_{ijm},y_{ijmk},Z_{k}\in \left\{0,1\right\},\forall m,i,k$$

Equation (9) indicates that each task can be assigned to one workstation at a time. Equation (10) shows there are no idle open workstations. Equation (11) represents the disassembly task that is ready to be assigned only when all its AND relationship predecessors have been assigned. Equation (12) represents the disassembly tasks that can be allocated when at least one OR relationship predecessor task is allocated. Equation (13) indicates the total uncertainty and processing time of all disassembly tasks assigned to the workstation must be processed within the cycle time. The decision variables are all 0–1 variables in Equation (14).

#### 3.3.3. The Lower Bound

The lower bound was first proposed by Gökçen et al. [47], which represents the theoretical minimum number of stations for balancing a parallel assembly line under certain conditions. The origin lower bound ($LB_{o}$) can be calculated as follows:

(15) $$LB_{o}=\left\lceil \sum_{m=1}^{M}\frac{\sum_{i=1}^{I_{m}}\mu_{im}}{CT_{m}}\right\rceil ,LB_{o}\in N^{+}$$

In Equation (15), $\mu_{im}$ is the deterministic factor representing the average disassembly time. When disassembly time is considered as a stochastic factor, refer to Özcan [45], then the lower bound (LB) for DLBP-SP is modified as Equation (16).

(16) $$LB=\left\lceil \sum_{m=1}^{M}\frac{\sum_{i=1}^{I_{m}}\mu_{im}+\varphi^{-1}\left(1-\alpha \right)\sqrt{\sum_{i=1}^{Im}\sigma_{im}^{2}}}{CT_{m}}\right\rceil ,LB\in N^{+}$$

In this equation, the disassembly time is considered stochastic, which obeys the standard normal distribution. In practical scenarios, the disassembly time of each task will be added because of uncertain conditions or interrupted factors (such as tool breakdown, components sticking, etc.). The original sum of disassembly time from the feasible assigned set of disassembly tasks to a workstation may exceed the cycle time of the workstation. Therefore, the confidence level (1−α) is adopted to represent the possibility of the sum of the stochastic disassembly time of the assigned set of disassembly tasks within the cycle time of the workstation. In this paper, consistent with Özcan [45], the confidence level (1−α) is taken as 0.9 and 0.975, respectively. In addition, the random variances of disassembly tasks are generated and categorized into low task variances ([0, ${\left(\mu_{im}/4\right)}^{2}$]) and high task variances ([0, ${\left(\mu_{im}/2\right)}^{2}$]) as the initial parameter for the following comparison experiments in Section 5.

### 3.4. The Explanatory Example

In this part, an explanatory example is carried out to illustrate the proposed mathematical model of DLBP-SP. According to the parallel disassembly line shown in Figure 2, the pre-defined cycle time of each disassembly line ($CT_{m}$) and the information about different EoL products are introduced in Table 2 and Table 3. The random variances of disassembly tasks are considered low task variances. Therefore, the cycle time (CT) and coefficient values ($\varepsilon_{m}$) can be calculated through the LCM method from Equations (1)–(4). The modified information of products A and B on parallel disassembly lines is represented in Table 4.

The lower bound of the number of workstations is calculated as three according to Equation (15). A feasible optimal disassembly sequence, task allocation and operating rate are represented in Table 5.

This disassembly sequence achieves the theoretical minimum number of workstations which can be regarded as one of the optimal solutions. However, this solution is not unique. When taking the operating rate into account as another optimisation objective, this solution may not be one of the optimal solutions. On the contrary, if both the number of workstations and operating rate are optimal, this solution can be regarded as one Pareto optimal solution. The multi-objective optimisation process should be applied with optimisation algorithms. The optimisation algorithms will be proposed in the following part.

## 4. The Proposed Hyper-Heuristic Algorithm for DLBP-SP

This section introduces the novel simulated annealing-based hyper-heuristic algorithm (HH). Firstly, the precedence constraints of EoL products are represented and encoded based on the precedence graph. Next, the operations procedure and framework of the proposed HH are explained in detail. Finally, the decoding process is introduced to represent the multi-objective optimisation results of HH.

### 4.1. Encoding Strategy

Multiple feasible disassembly sequences must satisfy the precedence constraints of EoL products. Referring to Bentaha et al. [48], the precedence graph is used to create the precedence matrix for meeting the requirement of precedence constraints and generating a feasible initial solution. The precedence matrix of EoL products is set using binary variables to represent the precedence constraints of components of EoL products. As shown in Equation (17), the precedence matrix of EoL product on the disassembly line m is $P_{m}$:

(17) $$P_{m}={\left[P_{ijm}\right]}_{\left(N_{m}\times N_{m}\right)},\forall i,j=1,2,\ldots ,N_{m};m=1,2,\ldots ,M$$

As shown in Equation (18), $P_{ijm}$ represents the precedence relationship between disassembly task *i* and task *j*. The equation must satisfy the decision variable:

(18) $$\begin{array}{cc} P_{ijm} & =\left\{\begin{array}{c} =1,\;\mathrm{if}\;\mathrm{task}\;i\;\mathrm{immediate}\;\mathrm{predecessor}\;\mathrm{of}\;\mathrm{task}\;j\\ =0,\;\mathrm{otherwise}\; \end{array}\right. \end{array}$$

Different EoL products will have different precedence constraints. In order to manage the precedence constraints of different EoL products in DLBP-SP, the composite precedence matrix is constructed to represent the relationships among different EoL products, as shown in Equation (19) [15]:

(19) $$P^{*}=\left[\begin{array}{ccccc} P_{1} & 0 & 0 & 0 & 0\\ 0 & \ldots & 0 & 0 & 0\\ 0 & 0 & P_{m} & 0 & 0\\ 0 & 0 & 0 & \ldots & 0\\ 0 & 0 & 0 & 0 & P_{M} \end{array}\right]$$

The composite precedence matrix $P^{*}$ is a diagonal matrix. The precedence matrices for different EoL products are assigned in a sequential manner and other elements are zero matrices in $P^{*}$.

**Figure 3:**
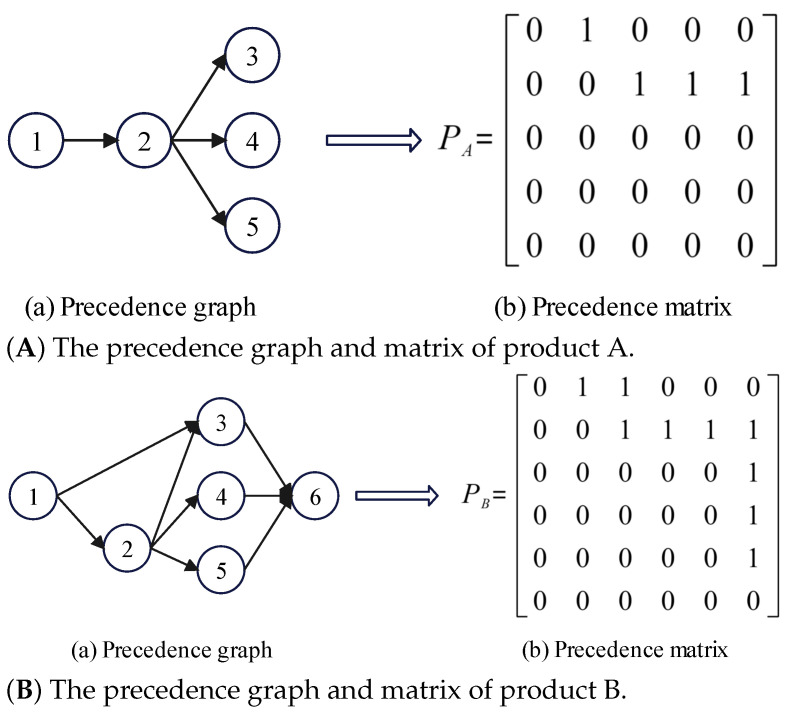
The precedence graph and matrix of product.

For example, based on the explanatory example, the precedence graph and precedence matrix of EoL products A and B are shown in Figure 3, respectively. The constructed composite precedence matrix $P^{*}$ is shown in Equation (20).

(20) $$P^{*}=\left[\begin{array}{cc} \left[\begin{array}{ccccc} 0 & 1 & 0 & 0 & 0\\ 0 & 0 & 1 & 1 & 1\\ 0 & 0 & 0 & 0 & 0\\ 0 & 0 & 0 & 0 & 0\\ 0 & 0 & 0 & 0 & 0 \end{array}\right] & 0\\ 0 & \left[\begin{array}{cccccc} 0 & 1 & 1 & 0 & 0 & 0\\ 0 & 0 & 1 & 1 & 1 & 1\\ 0 & 0 & 0 & 0 & 0 & 1\\ 0 & 0 & 0 & 0 & 0 & 1\\ 0 & 0 & 0 & 0 & 0 & 1\\ 0 & 0 & 0 & 0 & 0 & 0 \end{array}\right] \end{array}\right]$$

The composite precedence matrix is the input and premise for generating the initial solution of DLBP-SP. The process steps of determining the feasible disassembly sequence are described according to the explanatory example as follows:

Step 1: Determine the disassembly task that has no predecessor tasks as the priority disassembly tasks. The priority disassembly tasks should be $A_{1}$ or $B_{1}$.

Step 2: After all the priority disassembly tasks have been assigned, the composite precedence matrix should be updated. For example, before assigning task $A_{1}$, the origin $P^{*}\left(A_{1},A_{2}\right)=1$. After task $A_{1}$ is assigned, the updated ${P^{*}}^{\prime }\left(A_{1},A_{2}\right)=0$. In addition, the upper-left outer matrix becomes zero, which can be deleted during disassembly task sequencing.

Step 3: The disassembly tasks that do not have AND predecessor tasks or those with OR predecessor tasks are randomly selected as the following disassembly tasks, such as $A_{2}$ or $B_{2}$.

Step 4: To reach $P^{*}=\left[0\right]$, repeat step 2 and step 3. When $P^{*}=\left[0\right]$, all the disassembly tasks in DLBP-SP have already been assigned and sequenced. In the following explanatory example, Table 5 shows one disassembly sequence of the DLBP-SP. Constraints and components of products A and B are simple. With the larger scale of EoL products, the sequencing process becomes more complex.

### 4.2. Procedures of the Proposed Hyper-Heuristic Algorithm

Different from the general heuristic algorithm, the HH is an automated methodology which selects or generates heuristic algorithms suitable for solving multi-objective optimisation problems [49]. The typical framework of a hyper-heuristic algorithm can provide the chosen high-level heuristic algorithm (HLH) for managing a group of low-level heuristic algorithms (LLHs) to obtain the optimal solution [50].

#### 4.2.1. Low-Level Heuristic Algorithms

As the fundamental component of the framework, LLHs affect HH’s overall complexity and performance. Therefore, for designing the LLHs, the main principles of determining the LLHs should be relatively simple and have their own advantages for ensuring the overall performance of HH. There are three kinds of heuristic algorithms adopted as LLHs in this paper, including non-dominated sorting genetic algorithm 2 (NSGA2), strength Pareto evolutionary algorithm 2 (SPEA2), and multi-objective evolutionary algorithm based on decomposition (MOEAD). These LLHs have their own advantages and disadvantages.

1. NSGA2 [51]: adopts fast sorting and elite strategy for improving the convergence and accuracy of the algorithm and proposes the congestion degree for ensuring the variety and distribution of solutions. NSGA2 has good convergence for solving multi-objective optimisation problems. However, the distribution of the optimal solutions from NSGA2 is not uniform.
2. SPEA2 [52]: adopts the fine-grained fitness assignment strategy and density information that is suitable for solving multi-objective optimisation problems. SPEA2 has faster convergence and low computational complexity compared to the other two algorithms.
3. MOEAD [53]: transforms the multi-objective optimisation problem into multiple sub-scalar problems. Each sub-scalar problem is composed of the uniformly distributed weight vector and optimises each sub-scalar problem through an aggregation function to solve the multi-objective problems. However, the computational complexity of MOEAD is the highest among LLHs.

These three kinds of LLHs are relatively simple and suitable for utilisation as LLHs in HH. All the LLHs must apply the crossover and mutation processes to operate the initial solutions and generate the set of optimal solutions. This research introduces the partially mapped crossover and single-point insertion mutation for DLBP-SP.

#### 4.2.2. Partially Mapped Crossover

Typically, the random crossover method is one of the easiest methods for generating offspring solutions from optimal parent solutions. However, in DLBP-SP, the random crossover method can generate infeasible solutions that violate the precedence constraints and reduce the performance of the LLHs. Therefore, in this paper, the partially mapped crossover (PMX) method is used to improve the performance of LLHs.

**Figure 4:**
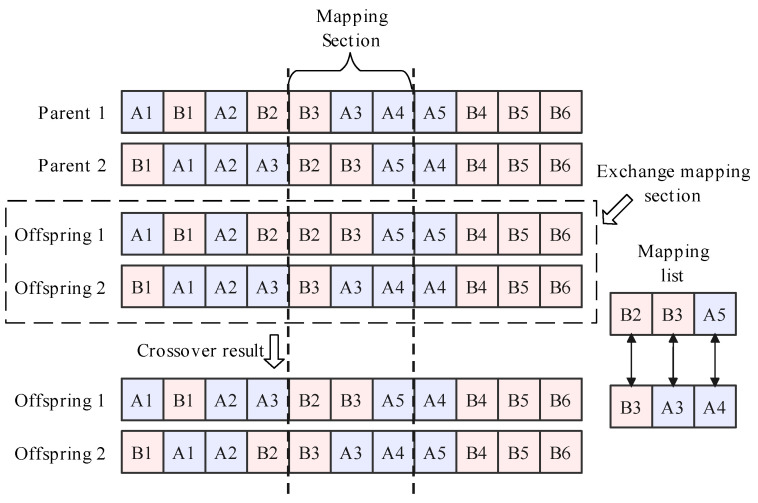
Operation process of the partial mapped crossover.

Taking the explanatory example, the operation process of partially mapped crossover is shown in Figure 4. The parents can be any two feasible solutions and the mapping section is determined between two random crossover points. During the exchange mapping section, the mapping list for exchanging is selected, for example, $B_{2}↔B_{2}↔A_{3},A_{5}↔A_{4}$. Next, conflict individuals should be updated according to the mapping list, and the rest of the non-conflict individuals can be copied directly from their parents. Finally, offspring solutions are generated.

#### 4.2.3. Single-Point Insertion Mutation

Similar to the crossover operation, the typical random mutation method is also easily generating infeasible solutions. Referring to Wang et al. [15], the single-point insertion mutation method is used for improving the performance of LLHs.

**Figure 5:**
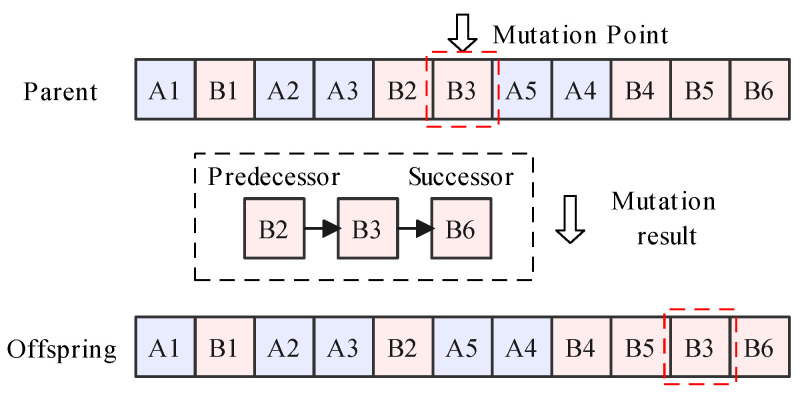
Operation process of single-point insertion mutation.

Taking the explanatory example, the operation process of single-point insertion mutation is shown in Figure 5. The mutation point is randomly selected, for example $B_{3}$. Then, the predecessor and successor tasks are determined ($B_{2}$ and $B_{6}$). Next, the selected point should be allocated after $B_{2}$ or before $B_{6}$. In the original parent solution, $B_{3}$ is immediately after $B_{2}$. Therefore, there is only one offspring solution that $B_{3}$ is assigned immediately before $B_{6}$.

### 4.3. Simulated Annealing Based High-Level Heuristic Algorithm

The HLH is the dominant component that directly affects the overall performance of HH. Selecting an appropriate high-level strategy is very important to solve optimisation problems. Currently, the HLHs are mainly divided into four categories according to different mechanisms, including random selection, greedy strategy, meta-heuristic algorithm and learning method [54]. This paper adopts the simulated annealing algorithm (SA) as the HLH in DLBP-SP.

The SA has a great ability to solve parallel complex multi-objective optimisation problems. The computational complexity is simple and has strong robustness and universality. However, the performance of the SA algorithm is sensitive to the initial value and pre-defined parameters. The convergence rate of SA is also relatively slow. Therefore, choosing SA as the HLH in HH can effectively avoid local optimal and achieve better global optimal solutions through managing the multiple solution spaces that are generated from LLHs. The procedure for the proposed SA-based HH is shown in Algorithm 1.
**Algorithm 1Proposed SA based HH.****Input:** Objective Function, *F*; Crossover, $p_{c}$; Mutation, $p_{m}$; Initial temperature, $T_{0}$; Stopping temperature, $T_{f}$; Cooling rate, α; Initial population, $P_{0}$; Iteration time, *K*; Precedence matrix, $P^{*}$; Population size, *N***Output:** Optimal solution set, $S_{*}$ 1:t←0 2:Random generate *N* individuals as the initial population 3:**while**t≤K or $S_{t}\neq S_{t-1}$ **do** 4:      **for** i=1 to *N* **do** 5:            Generate initial solution sets $S_{0}$ through mapping, crossover ($p_{c}$) and mutation ($p_{m}$) based on LLHs ($H_{i}$) 6:            **while** $T_{0}\geq T_{f}$ **do** 7:                  Randomly select a heuristic $h_{i}\in H_{i}$, combine $S_{0}$ to generate new solution sets through neighborhood mutation $S_{i}$, Calculate $\Delta E_{k}=F\left(S_{1}\right)-F\left(S_{0}\right)$ 8:                  **if** $\Delta E_{k}\geq 0$ **then** 9:                        $S_{*}=S_{1}$10:                 **else**11:                      generate a random number x∼U(0,1)12:                      **if** $x<exp(-\Delta E_{k}/t)$ **then**13:                            $S_{*}=S_{1}$14:                      **else**15:                            $S_{*}=S_{0}$16:                      **end if**17:                 **end if**18:            **end while**19:      **end for**20:      t=t+121:**end while**

### 4.4. Decoding Process

The decoding process is allocating the optimal sequenced disassembly tasks to workstations while within the cycle time of DLBP-SP. According to explanatory example and Table 5, through decoding process under certain condition, the number of workstations is three. The sequential disassembly tasks in each workstation are: $K_{1}=A_{1}\to B_{1}\to A_{2}$, $K_{2}=B_{2}\to B_{3}\to A_{3}\to A_{4}\to A_{5}$, $K_{3}=B_{4}\to B_{5}\to B_{6}$, respectively. According to the NP characteristic of DLBP-SP, the optimal solution is not identical.

## 5. Computational Experiments

This section introduces the comparison experiment and the case study. Firstly, the comparison experiment is carried out by comparing with existing algorithms through benchmark test datasets for validating the performance of the proposed HH. Then, a case study is proposed based on two types of industrial splitter gearboxes. The results are also analyzed and discussed in this section. The proposed HH is implemented in Python and runs on an Intel(R) Core(TM) i7-9700K CPU 3.6 GHz computer with 32 GB of RAM.

### 5.1. Comparison Experiment

The proposed HH algorithm is compared with the Özcan [45] proposed the tabu search algorithm (TS) for dealing with the stochastic parallel assembly line balancing problem and Wang et al. [15] proposed the genetic simulated annealing algorithm (GSA) for solving the partially parallel stochastic DLBP.

#### 5.1.1. Description of the Collected Dataset

The benchmark test datasets is offered from Özcan [45], which contain 16 different named datasets (such as Jaeschke, Jackson, etc.). Each named dataset contains different number of disassembly tasks, precedence constraints and the mean process time of each task. In order to applied for parallel disassembly line, the problems are generated from the datasets paired to themselves and with other datasets (such as Jaeschke-Jaeschke, Jackson-Jaeschke, etc.). There are 31 different experimental problems considered in this research. A total of 372 experiments are implemented with different indicators, including cycle times ($CT_{1}$, $CT_{2}$), the number of tasks ($N_{1}$, $N_{2}$), different task variances and confidence levels (0.9 and 0.975). The number of workstations (*N*) is the single optimisation goal in this computational experiment. The results of TS and GSA are collected form reference [15,45].

#### 5.1.2. Results and Analysis

According to the proposed computational experiments, the experiment results are represented and summarised in Table 6 and Table 7, respectively. The proposed HH algorithm achieves most identical solutions under low task variance without much promotion. The rates of HH obtaining identical solutions under low task variance with TS and GAS are 87.10%, 84.94% and 73.12%, 80.64%, respectively. However, the proposed HH algorithm can achieve great improvement and have better solutions under high task variance conditions. The rates of HH obtaining better solutions under high task variance than TS and GAS are 89.24%, 97.84% and 86.02%, 97.84%, respectively. According to the results, we can indicate that our proposed HH algorithm has a similar performance as the existing TS and GSA algorithms under low task variance conditions. Since the variety and search space of the mean disassembly time is relatively small under low task variance conditions, optimal solutions can easily be obtained. The proposed HH algorithm performs better under high task variance conditions due to a larger search space. Therefore, the superior performance of the proposed HH algorithm can be validated through the results.

Additionally, the gap percentage (%Gap) from LB ($LB=\frac{Min(K)-LB}{LB}$) is introduced to evaluate the effectiveness of the proposed HH algorithm. The lower %Gap represents the calculated outcomes closer to the theoretical lowest number of workstations, which can also represent the performance of the optimisation algorithm. According to the results in Table 7, the %Gap from LB for (1−α)=0.9 and (1−α)=0.975 under low task variance are 9.37% and 14.29%. In addition, the %Gap from LB for (1−α)=0.9 and (1−α)=0.975 under high task variance are 7.63% and 13.17%, respectively. Compared to TS and GSA, the %Gap from the proposed HH algorithm under low task variance is similar with slightly improvement. Under high task variance, the %Gap from the proposed HH algorithm reduces 9.2% under (1−α)=0.9 and 8.86% under (1−α)=0.975 compared with TS, respectively. Meanwhile, the %Gap from proposed HH algorithm reduces 10.88% under (1−α)=0.9 and 17.84% under (1−α)=0.975 improvement compared with GSA, respectively. The stability of the proposed HH can also be represented by the similar results under different variances.

Based on the computational experiment results, we can indicate that the proposed HH algorithm is validated and suitable for more complicated and changing situations.

### 5.2. Case Study

This subsection applies the proposed HH algorithm for multi-objective optimisation of DLBP-SP with two similar types of gearboxes from Hansa Tmp Co., Ltd. The superior performance of the proposed HH algorithm can be verified from the number of non-dominated solutions compared with LLHs (NSGA2, SPEA2, and MOEAD) and a basic simulated annealing (SA) algorithm. The superiority of the proposed HH algorithm can be validated through the number of the non-dominated solutions. The stability and robustness of the proposed HH algorithm can also be proved based on the hyper-volume index.

#### 5.2.1. Descriptions of the Gearboxes

The gearbox is one of the most common and typical types of industrial equipment. On the one hand, most failure types of splitter gearboxes are minor problems on one component, whereas the rest of the main components function well [55]. On the other hand, the connection mode of components in the gearbox is relatively simple, which theoretically allows complete disassembly without any destructive processes. Therefore, the gearboxes have great potential value through remanufacturing. There are two similar types of gearboxes considered in this case study, including splitter gearboxes series 85000 and 90000, as shown in Appendix A.

This research only focuses on and collects information related to the disassembly process. Therefore, detailed running parameters and data are not considered. The installation information about the splitter gearboxes is collected, including the type and quantity of components (representing as mean disassembly process time), operation time deviation and revenue from each disassembled component. The detailed bill of materials of these splitter gearboxes is shown in Appendix A.

**Figure 6:**
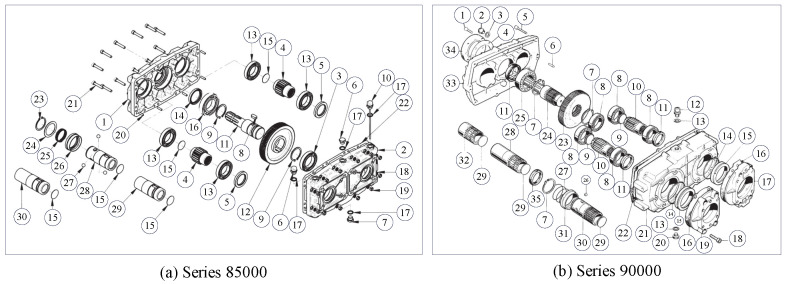
Explosion diagram of splitter gearboxes.

**Figure 7:**
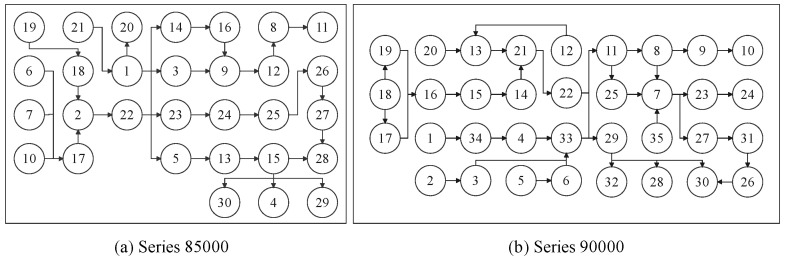
Precedence relationship of splitter gearboxes.

The splitter gearbox series 85000 has 30 parts, and the splitter gearbox series 90000 has 35 parts. The explosion diagram of both splitter gearboxes is represented in Figure 6, which are collected from open-source catalogue and presented in materials. The precedence constraints and disassembly sequence of splitter gearboxes series 85000 and 90000 are proposed according to the spare part list and the manufacturing process. The disassembly process can be regarded as the reverse process of the manufacturing process. The arrow direction shows the immediate precedence of each disassembly task. The splitter gearboxes are assumed as being able to be completely disassembled in this research. The precedence relationships for splitter gearboxes series 85000 and 90000 are proposed, as shown in Figure 7.

#### 5.2.2. Experiments and Analysis

The combinations of the different cycle times of each disassembly line are constructed in this subsection. The cycle times of each parallel disassembly line are set as $CT_{1}=\left\{8,27,50,60,90\right\}$ and $CT_{2}=\left\{10,24,60,65,108\right\}$, respectively. The combination of different experimental parameters is shown in Table 8.

The initial population for each algorithm is set as 50, and the crossover and mutation probability in related algorithms are set as 0.8 and 0.2, respectively. The initial temperature is set as 200, and the cooling rate is set as 0.975. The final minimum temperature is 10.

Table 9 presents three optimisation objectives and the number of non-dominated solutions from different experiments. When the cycle time of one disassembly line is determined, the number of workstations (*K*) is reduced while the cycle time of the other disassembly line is increasing. Aside from the number of workstations, the minimum workload smoothness index (*I*) and the maximum profit (*P*) are realised when the same cycle time ($CT_{1}=CT_{2}=60$) is achieved, which is a special scenario for parallel disassembly lines. However, there is no direct connection between *I* and *P*. There are several experiments where the results of *I* are similar, but the results of *P* are very different, such as experiments 7 and 8, 13 and 14. There are three experiments that generate costs, which are not suitable for taking into account. Moreover, the greater number of non-dominated solutions represents the better performance of the optimisation algorithm. According to the experiment results in Table 9, the proposed HH algorithm can obtain a greater number of non-dominated solutions, which achieves 24 times the greatest number of non-dominated solutions out of all 25 experiments.

**Figure 8:**
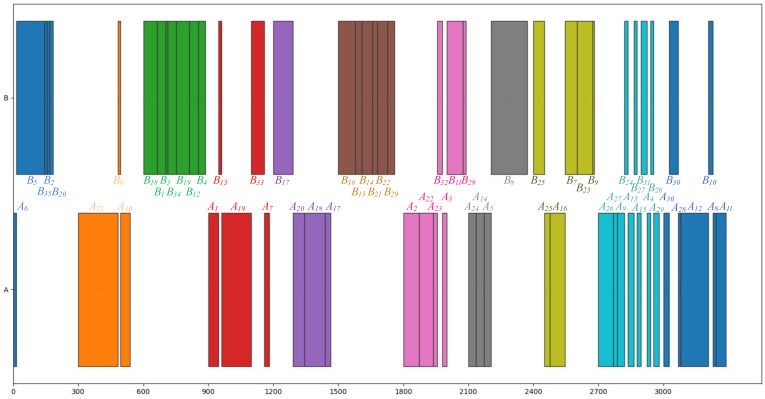
The Gantt chart of optimal solution ($CT_{1}=50,CT_{2}=60$).

Table 10, Table 11 and Table 12 are selected three schemes of the optimal solution based on different combinations of cycle time for detail analysis. Table 10 shows one optimal solution based on the $CT_{1}=50,CT_{2}=60$. Figure 8 intuitively shows the Gantt chart of the disassembly tasks allocation. The minimum number of workstations is 11, in which workstation 6 only works on disassembly line 1, whereas other workstations work on both disassembly lines. The working load balancing index is 124.12, the total profit from the disassembly process is 499.8, and the utilization rates of the workstation range from 96.1% to 61.4%. The utilization rates of the first two workstations are relatively low because there are some disassembly tasks that are time-consuming (such as tasks B5 and A21) and hard to achieve ideal results in a real-world application. Apart from those two workstations, the utilization rates of the rest nine workstations are above 85%.

Table 11 shows the one optimal solution based on the same cycle time $CT_{1}=CT_{2}=60$, which is a special situation that the cycle times of parallel disassembly lines are equal. The minimum number of workstations is 10, the working load balancing index is 19.87, the total profit from the disassembly process is 749.8, and the utilization rates of the workstation are all above 85.0%. The working load balancing index is extremely low as well as achieves the maximum revenue. This result is generated because the cycle time of each disassembly line is identical. All workstations have the best performance without conflict on each disassembly line under this circumstance.

Table 12 shows the one optimal solution based on the cycle times $CT_{1}=90,CT_{2}=108$, which is the largest combination in this case study. The minimum number of workstations is 6, the working load balancing index is 115.97, the total profit from the disassembly process is 309.8, and the utilization rate of the workstation ranges from 96.1% to 76.6%. Compared with the $CT_{1}=50,CT_{2}=60$, the working load balance has slightly decreased, while the overall profit has also declined. We can identify that the cycle time of each disassembly line is not the longer, the better. The longer cycle time will lead to a longer running time for workstations. However, the utilization rate of workstations may not increase. It is well worth determining the cycle time of each parallel disassembly line at the initial design stage based on the disassembly EoL products.

Moreover, this paper adopts the hypervolume index as an indicator for evaluating the performance of the proposed HH algorithm. The hypervolume index represents the volume of the hypercube enclosed by the individual points in the solution set and the reference point in the target space [56]. This hypervolume index is suitable for evaluating the convergence and distribution of the optimal solution set from multi-objective optimisation algorithms. The higher mean value and lower number of outliers of the hypervolume outcomes indicate better convergence and uniformity of the algorithm.

**Figure 9:**
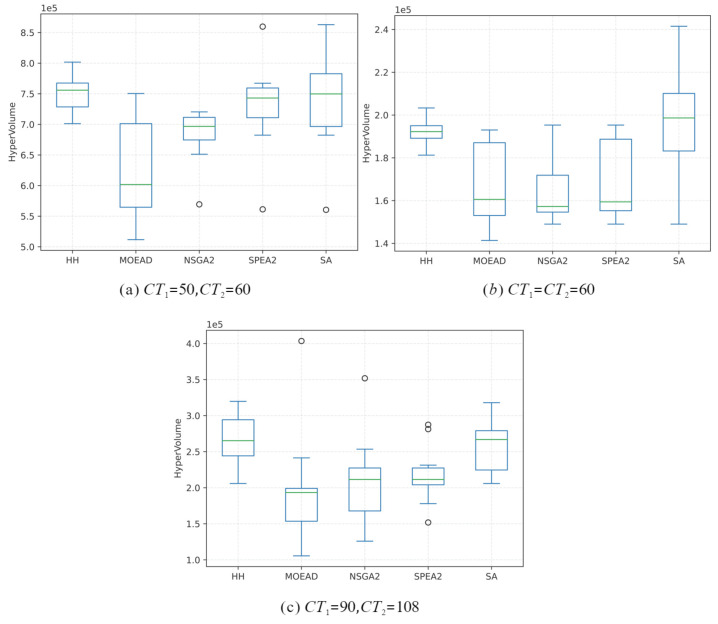
Box plots of hypervolume.

Based on the selected and analyzed three disassembly schemes above, three hypervolume index results are shown in Figure 9. According to the figures, the HH can obtain better results without deviant points in the HH solution sets. The results of low-heuristic algorithms are similar, which reflects that the performance gap of each low-heuristic algorithm is relatively small, and the basic SA algorithm has better performance than these low-heuristic algorithms. Convergence and distribution can be reflected in the distribution length of the box plot. The HH algorithm has the best performance of the convergence and uniformity of the distribution compared to the other four basic algorithms.

#### 5.2.3. Discussion

In summary, the validity and superiority of the proposed HH algorithm are verified through comparison with the tabu search algorithm and the genetic simulated annealing algorithm. Additionally, taking two types of splitter gearboxes as a case study, the HH algorithm is applied for multi-objective optimization of the disassembly of industrial equipment. The superior performance of the algorithm is verified by the number of non-dominated solutions. Based on the hyper-volume index, the HH algorithm is also shown to be stable and robust compared to low-level heuristic algorithms and the SA algorithm.

The main objective of this study is to improve the mathematical model and propose a methodology to optimise the DLBP-SP, which is the premise for designing and applying parallel disassembly lines for real-world applications. However, a limitation of the proposed approach is the lack of dynamic planning and real-time monitoring at this stage. Indeed, various advanced sensing technologies emerged in recent years can be implemented in the proposed parallel disassembly lines and integrated into our proposed approach to further improve their intelligence and efficiency. Mobile visual sensor systems, for example, can be integrated into parallel disassembly lines for real-time monitoring, which enables the detection of anomalies and failures for dynamic planning and further optimisation of the parallel disassembly lines [57]. Moreover, autonomous robotic, automated guided vehicle (AGV), and smart sensor devices have significant potential advantages when combined with parallel disassembly lines. For example, the virtual sensor network proposed by Indri et al. [58] can be integrated into the parallel disassembly lines for robot manual guidance and collision detection. Embedded with several types of physical sensors such as light beamers, vision sensors, and HD cameras, the network can provide real-time decision-making capability for the disassembly lines to further improve the overall production efficiency. Further investigations on the integration of various sensing technologies in the proposed approach will be conducted in our future work.

## 6. Conclusions

This paper originally proposed a novel simulated annealing-based hyper-heuristic algorithm to optimize the multi-objective stochastic parallel disassembly line balancing problem (DLBP-SP). Firstly, the mathematical model of stochastic parallel complete disassembly lines is developed for modelling the disassembly process in remanufacturing. This mathematical model is closer to real-world disassembly situations since the uncertain conditions of EoL products are represented by the stochastic disassembly time. Secondly, NSGA2, SPEA2 and MOEAD are taken into account as low-level heuristic algorithms and the simulated annealing algorithm as the high-level heuristic algorithm for combining the advantages of those basic algorithms. In addition, partially mapped crossover and single-point insertion mutations are used for ensuring precedence constraints and enhancing the quality of optimal solutions. Following that, single-objective optimisation computational experiments are used to verify the performance of the proposed HH algorithm. In the comparison experiment, the rates of HH obtaining identical solutions under low task variance are 87.10%, 84.94% and 73.12%, 80.64%, the rates of HH obtaining better solutions under high task variance are 89.24%, 97.84% and 86.02%, 97.84% compared with TS and GAS, respectively. Therefore, validity and superiority can be proved according to the comparison experiment results. Moreover, to our knowledge, this paper is the first to present gearboxes as a case study in DLBP. As discussed in Section 5.2.1, gearboxes are ideal products for complete disassembly without destructive processes. This research can be extended and integrated to design parallel disassembly lines for massive multi-types of gearboxes in remanufacturing systems. The multi-objective optimisation is carried out in a case study based on two types of gearboxes. The proposed HH algorithm achieves the greatest number of non-dominated solutions compared to the other four basic algorithms. In addition, the box plots of hypervolumn on the proposed HH algorithm realize the highest mean number and without any noise point. Through the case study results, better stability and convergence are demonstrated. The versatility of the proposed HH algorithm solution can also be proved from both single and multiple optimisation problems.

In future research, the mathematical model of disassembly line could be further improved to better model actual conditions of disassembly in remanufacturing. The collaborative human–robot disassembly line in a workstation will provide more alternative choices in disassembly task allocation, which makes it a more complex scenario. Moreover, the emerging deep learning and reinforcement learning optimisation algorithms may have more advantages for multi-objective optimisation problems. Deep learning based algorithms have great ability on solving nonlinear fitting, while reinforcement learning based algorithms are suitable for decision-making learning [59]. Nowadays, the deep reinforcement learning method is generated and combined the advantages of those two methods, which has great ability to solve more complex optimisation problems in real-world scenarios [60]. Due to the highly versatile of the proposed HH algorithm, it is possible to combine hyper-heuristic algorithms with those intelligence algorithms to further enhance the performance of optimisation problems. Additionally, the optimisation of DLBP in remanufacturing can be considered and pursued for larger-scale and more complex actual products.

## Figures and Tables

**Table 1:** Definition and description of notations.

Notations	Definition and Description
*m*	Number of disassembly line, *m* = 1, 2
$i_{m}$	Number of disassembly tasks on disassembly line m,i=1,2,…,I, where *I* is the number of components of EoL product
*K*	Number of workstations, k=1,2,…,K, where *K* is the maximum number of workstations
*j*	The position of the disassembly process, j=1,…,J, where *J* is the maximum number J=I.
$r_{i}$	Revenue from disassembly task *i*
$C_{w}$	Fix operation cost per unit time for workstations
$C_{p}$	Operating cost of workstations for parallel disassembly lines
$C_{c}$	Operating cost of workstations for single disassembly line
$CT_{m}$	Cycle time of disassembly line *m*
CT	Cycle time of parallel disassembly lines
$T_{k}$	Operation time of workstation *k*
$\varepsilon_{m}$	Coefficient value of CT and $CT_{m}$
${\tilde{t}}_{im}^{\prime }$	Stochastic disassembly time of task *i* on disassembly line *m*
$\mu_{im}$	Average disassembly time of task *i* on disassembly line *m*
$\sigma_{im}^{2}$	Variance of task *i* on disassembly line *m*
1−α	Confidence level
φ	Standard normal distribution function
LB	Theoretical minimum number of workstations
*I*	Workload smoothness index
*P*	Overall profit from complete disassembly process
$PAND(i_{m})$	The set of AND predecessors of task *i* on disassembly line *m*
$POR(i_{m})$	The set of OR predecessors of task *i* on disassembly line *m*
$x_{ijm}$	=$\left\{\begin{array}{c} =1,\;\mathrm{if}\;\mathrm{task}\;i\;\mathrm{at}\;\mathrm{position}\;j\;\mathrm{on}\;\mathrm{line}\;m\\ =0,\;\mathrm{otherwise}\; \end{array}\right.$
$y_{ijmk}$	=$\left\{\begin{array}{c} =1,\;\mathrm{if}\;\mathrm{task}\;i\;\mathrm{at}\;\mathrm{position}\;j\;\mathrm{on}\;\mathrm{line}\;m\;\mathrm{is}\;\mathrm{assigned}\;\mathrm{to}\;\mathrm{workstation}\;k\\ =0,\;\mathrm{otherwise}\; \end{array}\right.$
$S_{k}$	=$\left\{\begin{array}{c} =1,\;\mathrm{if}\;\mathrm{workstation}\;k\;\mathrm{is}\;\mathrm{working}\;\mathrm{on}\;\sin\mathrm{gle}\;\mathrm{disassembly}\;\mathrm{line}\;\\ =0,\;\mathrm{otherwise}\; \end{array}\right.$
$P_{k}$	=$\left\{\begin{array}{c} =1,\;\mathrm{if}\;\mathrm{workstation}\;k\;\mathrm{is}\;\mathrm{working}\;\mathrm{on}\;\mathrm{parallel}\;\mathrm{disassembly}\;\mathrm{line}\;\\ =0,\;\mathrm{otherwise}\; \end{array}\right.$
$Z_{k}$	=$\left\{\begin{array}{c} =1,\;\mathrm{if}\;\mathrm{workstation}\;k\;\mathrm{is}\;\mathrm{available}\;\\ =0,\;\mathrm{otherwise}\; \end{array}\right.$

**Table 2:** The information of product A on disassembly line 1.

Cycle Time of Disassembly Line 1 (${\mathrm{CT}}_{1}$)	15				
Task ID ($i_{1}$)	1	2	3	4	5
Average disassembly time ($\mu_{i1}$)	4	6	3	4	2
Variance ($\sigma_{i1}^{2}$)	0.50	1.20	0.70	0.60	0.20
Precedence constraints	-	1	1, 2	1, 2	1, 2

**Table 3:** The information of product B on disassembly line 2.

Cycle Time of Disassembly Line 2 (${\mathrm{CT}}_{2}$)	20					
Task ID ($i_{2}$)	1	2	3	4	5	6
Average disassembly time ($\mu_{i2}$)	3	4	2	6	7	4
Variance ($\sigma_{i2}^{2}$)	0.40	0.30	0.10	1.20	1.50	0.30
Precedence constraints	-	1	1, 2	1, 2, 3	1, 2	1, 2, 3, 4

**Table 4:** The modified information of product A and B on parallel disassembly lines.

CT = 60											
$\varepsilon_{1}$ = 4, $\varepsilon_{2}$ = 3											
TaskID	A1	A2	A3	A4	A5	B1	B2	B3	B4	B5	B6
$\mu_{i}^{\prime }$	16	24	12	16	8	9	12	6	18	21	12
$\sigma_{i}^{2\prime }$	8.00	19.20	11.20	9.60	3.20	3.60	2.70	0.90	10.80	13.50	2.70

**Table 5:** A feasible disassembly sequence of the DLBP-SP.

Number of Workstation	1			2					3		
Sequential task ID	A1	B1	A2	B2	B3	A3	A4	A5	B4	B5	B6
μ	16	9	24	12	6	12	16	8	18	21	12
*i*	8.00	3.60	19.20	2.70	0.90	11.2	9.60	3.20	10.80	13.50	2.70
Sum of	49			54					51		
Operating rate (%)	76.67			90.00					85.00		

**Table 6:** Computational results.

Problem	N1	N2	${\mathrm{CT}}_{1}$	${\mathrm{CT}}_{2}$	Low Task Variances								High Task Variance							
					(1−α)=0.9				(1−α)=0.975				(1−α)=0.9				(1−α)=0.975			
					LB	TS	GSA	HH	LB	TS	GSA	HH	LB	TS	GSA	HH	LB	TS	GSA	HH
Jaeschke–Jaeschke	9	9	10	14	7	8	8	8	8	10	9	9	7	11	10	**8**	8	13	13	10
			10	10	8	10	10	10	9	12	12	12	8	14	14	10	10	15	15	12
			18	10	7	7	7	7	7	8	8	8	7	9	9	8	8	11	11	9
Jackson–Jaeschke	11	9	10	14	8	9	9	9	8	11	10	10	8	11	11	10	8	13	13	11
			10	10	9	12	12	12	9	14	14	14	9	14	14	12	10	15	15	14
			21	18	5	5	5	5	5	6	6	6	6	6	6	6	6	7	7	6
Jackson–Jackson	11	11	10	13	9	11	11	11	9	12	12	12	9	11	11	11	9	14	13	12
			14	14	8	9	9	8	8	9	9	8	8	9	9	8	8	10	10	9
			21	14	7	7	7	7	7	7	7	7	7	7	7	7	7	8	8	7
Roszieg–Jackson	25	11	18	21	11	11	11	11	11	12	12	12	11	13	12	11	11	14	13	12
			21	21	10	10	10	10	10	11	10	10	10	11	10	10	10	12	12	11
			25	14	10	10	10	10	10	11	11	11	11	11	11	11	10	12	12	11
Roszieg–Roszieg	25	25	18	25	14	14	14	15	14	15	15	15	14	16	16	15	14	17	17	16
			21	21	13	15	14	15	14	16	15	15	14	16	16	15	14	18	17	16
			32	25	11	11	11	11	11	11	11	11	11	11	11	11	11	13	12	11
Sawyer–Roszieg	30	25	41	32	13	15	14	14	13	15	15	15	14	16	15	15	14	17	17	15
			47	25	14	14	14	14	14	15	15	15	14	16	15	14	14	18	17	15
			54	21	13	14	14	14	14	15	15	15	14	16	15	14	14	18	17	15
Sawyer–Sawyer	30	30	36	41	18	21	20	21	18	22	22	22	19	24	22	21	19	27	26	23
			36	36	19	22	22	23	19	24	24	25	20	25	24	23	20	28	28	25
			75	54	11	12	12	12	11	13	13	13	12	13	13	12	12	14	14	13
Gunther–Sawyer	35	30	61	75	14	15	15	15	14	16	16	16	14	17	16	15	14	19	18	16
			69	54	14	16	15	16	14	17	17	17	15	18	17	16	15	20	19	17
			81	36	16	19	18	19	16	20	19	20	17	21	20	19	17	24	23	20
Gunther–Gunther	35	35	61	69	17	19	19	19	17	20	20	20	17	22	21	19	17	25	24	21
			69	69	16	18	17	18	16	19	19	19	16	20	20	18	16	24	22	19
			81	61	15	17	17	17	16	18	18	18	16	20	19	18	16	23	22	19
Kilbridge–Gunther	45	35	79	81	14	15	15	15	15	16	16	16	15	17	17	15	15	19	19	16
			69	69	17	18	18	18	17	19	19	19	17	20	19	18	17	22	22	20
			184	61	12	13	13	13	12	14	14	14	13	15	15	13	13	17	16	14
Kilbridge–Kilbridge	45	45	79	184	11	12	12	12	12	12	12	12	12	12	12	12	12	13	13	12
			92	92	13	14	14	14	14	15	15	15	14	15	15	14	14	16	16	15
			138	110	10	10	10	10	11	11	11	11	11	11	11	11	11	12	12	11
Hahn-Kilbridge	53	45	2338	92	13	14	14	14	14	15	15	15	14	15	15	**14**	14	16	16	**15**
			2004	69	16	18	18	18	17	19	19	19	17	19	19	**18**	17	21	21	**19**
			2338	184	10	10	10	11	11	11	11	11	11	11	11	11	11	12	12	**11**
Hahn-Hahn	53	53	2004	4676	11	12	12	12	11	13	13	13	12	13	13	**12**	12	14	14	**13**
			2806	2806	11	12	12	12	11	13	12	12	12	13	12	12	12	14	15	**13**
			4676	3507	8	8	8	8	8	9	8	8	9	9	9	9	9	9	9	**9**
Tonge-Hahn	70	53	293	2004	20	22	22	23	16	24	24	24	21	25	25	**23**	21	28	27	**24**
			410	2806	14	13	13	16	14	14	14	17	15	14	14	16	15	16	16	17
			468	3507	12	13	13	13	12	14	14	14	13	14	14	**13**	13	16	16	**14**
Tonge-Tonge	70	70	364	410	19	21	21	21	19	22	22	22	19	23	23	**21**	19	26	26	**22**
			468	468	16	17	17	17	16	18	18	18	16	19	19	**17**	16	21	21	**18**
			527	293	19	22	22	22	19	23	23	23	20	24	24	**22**	20	27	27	**23**
Wee-Mag-Tonge	75	70	50	320	42	55	50	55	42	63	62	64	43	63	62	**56**	43	71	67	**65**
			52	364	40	48	45	48	40	57	55	57	40	60	56	**54**	40	66	62	**61**
			54	527	35	42	40	41	36	49	44	48	35	54	48	**41**	36	58	56	**53**
Wee-Mag-Wee-Mag	75	75	50	56	57	77	67	77	57	95	90	95	58	103	98	**79**	59	113	109	**98**
			52	52	58	82	74	81	58	104	103	105	60	107	104	**82**	60	113	112	**107**
			56	54	54	67	65	67	55	83	76	82	55	97	91	**69**	57	108	106	**85**
Arcus83-Wee-Mag	83	75	5048	50	45	59	54	58	46	67	63	65	47	70	63	**60**	47	74	72	**68**
			5408	54	42	50	49	50	42	56	55	56	43	62	60	**51**	44	70	69	**58**
			5853	56	39	47	46	47	39	51	49	51	40	58	54	**48**	41	66	61	**51**
Arcus83-Arcus83	83	83	5048	5408	29	34	34	34	29	36	35	36	31	38	37	**34**	31	43	42	**36**
			6883	6883	22	25	25	25	22	26	26	26	24	28	28	**25**	24	31	31	**27**
			8898	6309	20	23	23	23	20	24	24	24	22	26	26	**24**	22	29	29	**25**
Lutz3-Arcus83	89	83	110	6309	29	31	31	31	29	33	33	33	29	35	35	**31**	29	39	38	**33**
			127	7571	25	27	26	27	25	28	28	28	25	29	29	**27**	25	32	32	**28**
			150	8898	21	22	22	22	21	23	23	23	21	24	24	**22**	21	27	27	**23**
Lutz3-Lutz3	89	89	110	150	28	30	30	30	28	32	32	32	28	33	33	**31**	28	37	37	**32**
			118	118	30	33	33	33	30	35	34	35	30	37	36	**33**	30	41	40	**35**
			137	127	27	29	29	29	27	31	31	31	27	32	32	**29**	27	36	36	**31**
Mukherje-Lutz3	94	89	301	137	28	30	30	30	28	32	32	32	28	33	33	**31**	28	37	37	**32**
			324	118	29	31	31	31	29	33	33	33	29	35	35	**32**	29	39	38	**34**
			351	150	25	26	27	**26**	25	28	28	28	25	29	29	**27**	25	32	32	**28**
Mukherje-Mukherje	94	94	301	301	29	33	33	33	29	35	35	35	30	36	36	**33**	30	40	40	**35**
			301	351	27	30	30	30	27	32	32	32	28	33	33	**30**	28	37	37	**32**
			351	324	26	29	29	29	26	31	31	31	27	32	32	**29**	27	36	35	**31**
Arcus111-Mukherje	111	94	8847	301	32	36	36	36	32	39	38	39	33	40	40	**37**	33	45	45	**39**
			9400	324	30	34	34	34	30	36	36	36	31	38	37	**34**	31	42	42	**36**
			10,027	351	28	31	31	31	28	33	33	33	29	35	34	**32**	29	39	38	**33**
Arcus111-Arcus111	111	111	8847	9400	34	39	39	39	34	42	41	42	35	44	43	**40**	35	49	48	**42**
			11,378	11,378	28	31	31	31	28	33	32	33	28	33	33	**31**	28	37	37	**33**
			17,067	10,743	23	26	26	26	23	28	28	28	24	29	29	**26**	24	32	32	**28**
Bartholdi-Arcus111	148	111	564	11,378	25	26	26	26	25	28	28	28	25	29	29	**26**	25	31	31	**28**
			705	11,570	22	24	24	24	22	25	25	25	23	26	26	**24**	23	28	28	**25**
			805	7571	28	31	31	31	28	33	33	33	28	35	34	**31**	28	37	38	**33**
Bartholdi-Bartholdi	148	148	513	564	22	23	24	**23**	22	24	25	24	23	25	25	**23**	23	27	28	**24**
			626	626	19	20	20	20	19	21	21	21	20	21	22	**20**	20	23	23	**21**
			805	705	16	17	17	17	16	17	17	17	17	18	18	**17**	17	19	19	**18**
Lee-Bartholdi	205	148	1510	564	26	28	29	**28**	26	30	30	30	27	31	31	**28**	27	34	34	**30**
			2077	626	21	22	23	**22**	21	23	23	23	22	24	24	**22**	22	26	26	**23**
			2832	705	17	18	18	18	17	19	19	19	18	19	19	**18**	18	20	21	**19**
Lee-Lee	205	205	1699	2643	23	25	25	25	23	26	26	26	23	27	27	**25**	23	29	29	**26**
			2266	2266	22	23	23	23	22	23	24	24	22	24	24	**23**	22	26	26	**24**
			2832	2454	19	19	20	20	19	20	20	20	19	21	21	**20**	19	22	22	**21**
Scholl-Lee	297	205	1935	2831	46	50	50	50	46	52	52	52	46	54	54	**50**	46	60	60	**52**
			2247	1699	46	50	50	50	46	53	53	53	46	54	54	**50**	46	60	60	**52**
			2787	1510	42	45	45	45	42	47	47	47	42	49	49	**45**	42	53	53	**47**
Scholl-Scholl	297	297	2049	2680	62	68	67	67	67	71	71	71	62	73	73	**68**	62	81	81	**71**
			2111	2111	68	75	75	75	68	78	78	78	68	82	81	**75**	68	90	90	**79**
			2787	2247	58	63	63	63	58	66	66	66	58	68	68	**63**	58	75	74	**66**

**Table 7:** Analysis of the computational results.

Computational Results Analysis	VS TS				VS GAS			
	Low Task Variance		High Task Variance		Low Task Variance		High Task Variance	
	(1−α)=0.9	(1−α)=0.975	(1−α)=0.9	(1−α)=0.975	(1−α)=0.9	(1−α)=0.975	(1−α)=0.9	(1−α)=0.975
Number of better solutions	6	9	83	91	5	1	80	91
Number of identical solutions	81	79	9	1	68	75	12	1
Number of worse solutions	6	5	1	1	20	17	1	1
Rate of better solutions (%)	6.45%	9.68%	89.24%	97.84%	5.38%	1.08%	86.02%	97.84%
Rate of identical solutions (%)	87.10%	84.94%	9.68%	1.08%	73.12%	80.64%	12.90%	1.08%
Rate of worse solutions (%)	6.45%	5.38%	1.08%	1.08%	21.50%	18.28%	1.08%	1.08%
%Gap of TS and GAS	9.67	14.37	16.83	22.03	9.51	16.98	18.51	31.01
%Gap	9.37	14.29	7.63	13.17	-			

**Table 8:** The combination of experiment parameters.

No.	${\mathrm{CT}}_{1}$	${\mathrm{CT}}_{2}$	No.	${\mathrm{CT}}_{1}$	${\mathrm{CT}}_{2}$	No.	${\mathrm{CT}}_{1}$	${\mathrm{CT}}_{2}$	No.	${\mathrm{CT}}_{1}$	${\mathrm{CT}}_{2}$
1	8	11	8	27	65	15	39	89	22	84	24
2	8	24	9	27	72	16	67	11	23	84	65
3	8	65	10	27	89	17	67	24	24	84	72
4	8	72	11	39	11	18	67	65	25	84	89
5	8	89	12	39	24	19	67	72			
6	27	11	13	39	65	20	67	89			
7	27	24	14	39	72	21	84	11			

**Table 9:** Multi-objective optimisation results and the number of non-dominated solutions.

No.	${\mathrm{CT}}_{1}$	${\mathrm{CT}}_{2}$	*K*	*I*	*P*	MOEAD	SPEA2	NSGAII	SA	HH
1	8	10	51	781.8	359.8	6	6	7	6	7
2	8	24	39	421.3	495.8	5	5	6	5	6
3	8	60	31	1866.6	479.8	6	6	7	7	7
4	8	65	31	8127.6	79.8	6	6	7	7	7
5	8	108	29	3259.8	403.8	7	7	8	7	7
6	27	10	38	3991.9	259.8	8	8	8	8	8
7	27	24	25	620.2	443.8	5	5	5	5	5
8	27	60	16	619.8	209.8	9	9	9	9	10
9	27	65	15	1767.9	−995.2	10	10	11	11	11
10	27	108	13	102.9	671.8	6	6	7	6	7
11	50	10	33	681.2	529.8	6	6	6	6	6
12	50	24	20	1505.8	109.8	5	5	5	5	5
13	50	60	11	124.1	499.8	7	7	8	7	8
14	50	65	10	127.9	159.8	6	6	8	7	8
15	50	108	8	486.8	−1870.2	9	9	10	10	10
16	60	10	32	803.4	529.8	6	6	6	6	6
17	60	24	19	290.9	599.8	3	3	3	3	3
18	60	60	10	19.9	749.8	11	10	10	11	11
19	60	65	9	142.6	39.8	6	6	7	6	7
20	60	108	7	46.6	299.8	3	3	3	3	3
21	90	10	30	1154.6	519.8	6	6	7	6	7
22	90	24	17	797.4	379.8	6	6	6	6	6
23	90	60	8	25.3	649.8	6	6	7	6	7
24	90	65	8	256.3	−340.2	6	6	7	6	7
25	90	108	6	116.0	309.8	10	10	10	10	10
Best number in 25 times						8	7	22	12	24
Rate (%)						32	28	88	48	96

**Table 10:** One optimal sequence of the disassembly process ($CT_{1}=50,CT_{2}=60$).

Workstation No.	Working Load Balance	Profit	Time	(%)	Task Sequence on Each Workstation
1	124.12	499.8	184.2	61.4%	‘A6’→‘B5’→‘B35’→‘B2’→‘B20’
2			239.0	79.7%	‘A21’→‘B6’→‘A10’
3			287.5	95.8%	‘B18’→‘B1’→‘B3’→‘B34’→‘B19’→‘B12’→‘B4’
4			281.0	93.7%	‘A1’→‘B13’→‘A19’→‘B33’→‘A7’
5			265.5	88.5%	‘B17’→‘A20’→‘A18’→‘A17’
6			260.0	86.7%	‘B16’→‘B15’→‘B14’→‘B21’→‘B22’→‘B29’
7			288.8	96.3%	‘A2’→‘A22’→‘A23’→‘B32’→‘A3’→‘B11’→‘B28’
8			271.5	90.5%	‘A24’→‘A14’→‘A5’→‘B8’
9			279.8	93.2%	‘B25’→‘A25’→‘A16’→‘B7’→‘B23’→‘B9’
10			279.2	93.0%	‘A26’→‘A27’→‘A9’→‘B24’→‘A13’→‘B27’→‘A15’→‘B31’→‘A4’→‘B26’→‘A29’
11			289.0	96.3%	‘A30’→‘B30’→‘A28’→‘A12’→‘B10’→‘A8’→‘A11’

**Table 11:** One optimal sequence of the disassembly process ($CT_{1}=CT_{2}=60$).

Workstation No.	Working Load Balance	Profit	Time	(%)	Task Sequence on Each Workstation
1	19.87	749.8	53.0	88.3%	‘A19’→‘B35’→‘B2’→‘A6’→‘A18’→‘B1’
2			56.6	94.3%	‘B5’→‘A21’
3			44.6	89.2%	‘A1’→‘B20’→‘A20’→‘A7’→‘A10’→‘B18’
4			57.8	96.3%	‘B17’→‘B19’→‘B16’→‘B3’→‘B6’→‘B34’
5			57.6	96.0%	‘A17’→‘A2’→‘B4’→‘B12’→‘B15’→‘A22’→‘A14’→‘B13’
6			51.0	85.0%	‘A23’→‘B3’→‘B14’→‘B21’→‘A5’→‘A24’→‘A13’→‘A3’→‘A15’
7			55.4	92.3%	‘A16’→‘A4’→‘A9’→‘A25’→‘A29’→‘A30’→‘A12’→‘A8’
8			58.4	97.3%	‘B22’→‘B11’→‘B29’→‘A26’→‘B28’→‘A27’→‘B25’
9			49.2	82.0%	‘A28’→‘B32’→‘B8’→‘B9’→‘A11’
10			53.8	89.7%	‘B7’→‘B27’→‘B31’→‘B10’→‘B23’→‘B24’→‘B26’→‘B30’

**Table 12:** One optimal sequence of the disassembly process ($CT_{1}=90,CT_{2}=108$).

Workstation No.	Working Load Balance	Profit	Time	(%)	Task Sequence on Each Workstation
1	115.97	309.8	490.9	90.9%	‘B5’→‘A19’→‘B12’→‘B6’→‘B1’→‘A7’→‘A10’→‘B18’
2			508.1	94.1%	‘B17’→‘A21’→‘A18’→‘A1’→‘B34’→‘B20’→‘B2’→‘B13’→‘B35’
3			512.2	94.9%	‘B4’→‘B19’→‘B16’→‘B3’→‘A6’→‘A20’→‘A17’→‘B15’→‘B14’→‘B33’→‘A2’→‘B21’
4			519.1	96.1%	‘B22’→‘B29’→‘B11’→‘B28’→‘B25’→‘A22’→‘B8’→‘A14’→‘B9’→‘B32’
5			481.4	89.1%	‘A16’→‘B10’→‘A3’→‘B7’→‘B23’→‘A9’→‘B24’→‘A23’→‘B27’→‘A24’→‘A25→‘A26→‘A5’
6			413.8	76.6%	‘A12’→‘A13’→‘B31’→‘A15’→‘B26’→‘B30’→‘A30’→‘A8’→‘A11’→‘A29’→‘A27’→‘A4’→‘A28’

## Data Availability

The data for Section 5.1 comparison experiments are cited from open-source ‘Balancing stochastic parallel assembly lines’. These data can be found here https://doi.org/10.1016/j.cor.2018.05.006 (accessed on 8 May 2018).

## Appendix A. Bill of Materials of Splitter Gearboxes

**Table A1:** Bill of materials of Splitter Gearboxes Series 85000.

No.	Description (Parts)	Quality (Q)	Mean Process Time (t)	Deviation (D)	Revenue (r)
1	Housing	1	8.2	2.1	25.3
2	Cover	1	10.4	3.5	43.5
3	Bearing 6010	1	5.6	1.2	12.6
4	Pinion gear	2	3.4	1.4	6.6
5	Sealing ring 45 × 65 × 8	2	7.6	2.2	4.8
6	Oil plug 3/8${}^{\prime \prime }$	2	4.8	2.0	2.2
7	Oil drain plug 3/8${}^{\prime \prime }$	1	5.2	1.6	1.4
8	Key 12*25	1	2.6	0.8	0.7
9	Snap ring UNI 7435-50	2	6.4	4.2	4.7
10	Oil dipstick with vent	1	7.3	1.4	2.6
11	Male P.T.O. shaft 1${}^{\prime \prime }$3/8 Z6	1	8.4	3.4	23.4
12	Ring gear	1	18.7	5.2	4.3
13	Bearing 6009	4	5.4	1.3	11.2
14	Sealing ring 50*65*8	1	4.7	1.4	2.5
15	Cap DIN 470 D.38	5	10.5	4.5	1.5
16	Bearing 6210	1	10.2	3.5	15.6
17	Gasket	4	4.8	1.6	60.4
18	Washer Grower d.8	12	15.6	1.2	67.9
19	Nut M8	12	25.2	2.4	7.2
20	Peg UNI 8751 6*24	8	10.4	1.6	0.8
21	Socket cap screw M8*45	12	27.6	3.6	42.2
22	Gasket	1	8.5	1.4	14.3
23	Snap ring UNI 7435-48	1	3.4	1.2	2.3
24	Ring	1	4.7	2.1	3.7
25	Spring	1	2.6	1.4	2.3
26	Spring ring	1	8.6	2.4	4.2
27	Ball	3	4.2	0.9	12.7
28	Female P.T.O. shaft—1${}^{\prime \prime }$3/8${}^{\prime \prime }$ Z6	1	4.6	1.6	16.6
29	Female P.T.O. shaft short 1${}^{\prime \prime }$3/8	1	5.2	1.4	20.7
30	Female P.T.O. shaft long 1${}^{\prime \prime }$3/8	1	3.4	0.8	23.4

**Table A2:** Bill of materials of Splitter Gearboxes Series 90000.

No.	Description (Parts)	Quality (Q)	Mean Process Time (t)	Deviation (D)	Revenue (r)
1	Socket cap screw M6×20	4	9.2	1.2	14.2
2	Oil level plug	1	2.4	1.1	1.4
3	Gasket	1	1.2	0.4	0.6
4	Gasket	1	6.5	2.4	1.4
5	Socket cap screw	10	23.1	6.4	31.2
6	Peg ø 6	2	2.6	0.6	0.2
7	Snap ring ø 58	3	9.6	3.2	5.4
8	Bearing type 6010	5	28	6	63
9	Cap DIN 470	2	4.2	1.8	0.6
10	Pinion Gear	2	3.4	1.4	6.6
11	Sealing ring ø	3	11.4	2.1	7.2
12	Oil dipstick with vent	1	7.3	1.4	2.6
13	Gasket	3	3.6	1.2	45.3
14	O-Ring	2	8.2	2.2	0.6
15	Corteco Ring	2	10.4	3.8	6.4
16	Gasket	2	16.4	6.4	23.6
17	Flange SAE B	1	12.7	4.2	16.6
18	Socket cap screw	6	13.8	1.8	21.1
19	Flange SAE A	1	14.2	4.1	23.5
20	Oil drain plug 3/8${}^{\prime \prime }$	1	5.2	1.6	1.4
21	Housing	1	8.4	2.2	25.4
22	Gasket	1	8.5	1.4	14.3
23	Ring gear	1	18.7	5.2	4.3
24	Male P.T.O. shaft 1${}^{\prime \prime }$3/8	1	4.6	1.6	16.4
25	Bearing type 6210	1	10.2	3.5	15.6
26	Ball	3	4.2	0.9	12.7
27	Spring	1	2.6	1.4	2.3
28	Female P.T.O. shaft 1${}^{\prime \prime }$3/8 long	1	3.4	0.8	23.4
29	Cap DIN 470	3	6.3	2.7	0.9
30	Female P.T.O. shaft 1-3/8${}^{\prime \prime }$	1	7.3	2.4	18.4
31	Spring ring	1	8.6	2.4	4.1
32	Female P.T.O. shaft 1${}^{\prime \prime }$3/8 short	1	5.2	1.4	20.7
33	Cover	1	10.8	2.4	24.8
34	Cap	1	8.6	2.2	8.2
35	Ring	1	4.8	1.8	3.8
